# The Dopamine Allosteric Agent, PAOPA, Demonstrates Therapeutic Potential in the Phencyclidine NMDA Pre-clinical Rat Model of Schizophrenia

**DOI:** 10.3389/fnbeh.2018.00302

**Published:** 2018-12-12

**Authors:** Ritesh P. Daya, Jayant Bhandari, Sharnpreet K. Kooner, Joella Ho, Christopher D. Rowley, Nicholas A. Bock, Troy Farncombe, Ram K. Mishra

**Affiliations:** ^1^Department of Psychiatry and Behavioural Neurosciences, McMaster University, Hamilton, ON, Canada; ^2^Department of Psychology, Neuroscience and Behaviour, McMaster University, Hamilton, ON, Canada; ^3^Department of Radiology, McMaster University, Hamilton, ON, Canada

**Keywords:** allosteric modulator, dopamine, NMDA, phencyclidine, rat model, schizophrenia, PET imaging, pre-clinical

## Abstract

PAOPA, a potent analog of prolyl-leucyl-glycinamide, has shown therapeutic potential at the preclinical stage for dopaminergic related illnesses, including animal models of schizophrenia, Parkinson’s disease and haloperidol-induced extrapyramidal movement disorders. PAOPA’s unique allosteric mechanism and dopamine D2 receptor specificity provide a unique composition of properties for the development of potential therapeutics for neuropsychiatric illnesses. We sought to investigate PAOPA’s therapeutic prospects across the spectrum of schizophrenia-like symptoms represented in the established phencyclidine-induced rat model of schizophrenia, (5 mg/kg PCP twice daily for 7 days, followed by 7 days of drug withdrawal). PAOPA was assessed for its effect on brain metabolic activity and across a battery of behavioral tests including, hyperlocomotion, social withdrawal, sensorimotor gating, and novel object recognition. PAOPA showed therapeutic efficacy in behavioral paradigms representing the negative (social withdrawal) and cognitive-like (novel object recognition) symptoms of schizophrenia. Interestingly, some behavioral indices associated with the positive symptoms of schizophrenia that were ameliorated in PAOPA’s prior examination in the amphetamine-sensitized model of schizophrenia were not ameliorated in the PCP model; suggesting that the deficits induced by amphetamine and PCP—while phenotypically similar—are mechanistically different and that PAOPA’s effects are restricted to certain mechanisms and systems. These studies provide insight on the potential use of PAOPA for the safe and effective treatment of schizophrenia.

## Introduction

Prolyl-leucyl-glycinamide (PLG), a cleaved tripeptide of the oxytocin molecule and a dopamine allosteric modulator, has shown therapeutic promise in clinical studies of neuropsychiatric illnesses, including major depressive disorder (Ehrensing and Kastin, 1978; Kastin et al., 1980; Ehrensing et al., 1994). Our group has synthesized over 200 analog molecules of PLG. Of these, (3R)-2-Oxo-3-[[(2S)-2-Pyrrolidinylcarbonyl]amino]-1-pyrrolidineacetamide (PAOPA) has demonstrated more potent positive allosteric activity for the dopamine D2/D4 receptors compared to PLG (Verma et al., 2005). PAOPA’s interaction with the dopamine D2 receptor has been well characterized, including its distinct effect on G-protein coupled regulatory and downstream proteins (Mishra et al., 1990, 1997, 1999; Basu et al., 2013).

As a unique dopaminergic agent, PAOPA has been investigated for its treatment efficacy in animal models of Parkinson’s disease, movement disorders such as tardive dyskinesia, and schizophrenia. In the 6-hydroxydopamine lesioned rat model and 1-methyl-4-phenyl-1,2,3,6-tetrahydropyridine (MPTP)-induced mouse model of Parkinson’s disease, PAOPA modulated rotational behavior and showed neuroprotective effects, respectively (Mishra et al., 1997; Marcotte et al., 1998). PAOPA also prevented haloperidol-induced movement abnormalities in rats displaying a tardive dyskinesia-like state (Sharma et al., 2003).

PAOPA has ameliorated biochemical and behavioral abnormalities specific to the positive- and negative-like symptoms of schizophrenia in pre-clinical models. In the amphetamine sensitized animal model of schizophrenia, PAOPA showed remarkable effects in treating and preventing amphetamine-induced sensorimotor gating deficits, hyperlocomotion (akin to the positive-like symptoms) and social interaction deficits (akin to the negative-like symptoms) (Beyaert et al., 2013). In the MK-801 induced model of schizophrenia, PAOPA ameliorated social deficits, a phenotype modeling aspects of the negative symptoms of schizophrenia, (Dyck et al., 2011). These previous studies also describe its potential treatment efficacy in the prodromal stage and during illness. With these findings considered, PAOPA appears to be a viable candidate for the potential treatment of schizophrenia. However, its therapeutic efficacy for the cognitive symptoms of schizophrenia—a critical and under-treated symptom domain—remains to be investigated.

To date, NMDA receptor antagonists present one of the most acknowledged pre-clinical models of the negative and cognitive symptoms when administered in particular regimens to animals (Neill et al., 2010; Meltzer et al., 2013; Reynolds and Neill, 2016). These models are currently the most valid and accepted with which to examine the therapeutic index of potential drug candidates for schizophrenia (Large, 2007; Steeds et al., 2015). Phencyclidine (PCP) is a non-competitive antagonist for the NMDA receptor and induces schizophrenia-like behavioral abnormalities in humans and rodents. PCP has been shown to alter glutamatergic, GABAergic, and dopaminergic neurotransmission. In rodents, various PCP administration regimens exist to model different facets of schizophrenia (Neill et al., 2010).

In this study, we examined the therapeutic potential of PAOPA in a 7-day, bi-daily PCP treatment regimen followed by a 1-week drug withdrawal period. This particular regimen has proven to be the most robust in modeling novel object recognition impairments and social interaction deficits (Meltzer et al., 2013; Rajagopal et al., 2014). Sub-chronic PCP treatment has also been shown to produce a multi-system neurochemical imbalance, akin to the multi-system dysfunction observed in schizophrenia (Jentsch and Roth, 1999). A 1-week drug withdrawal period also allows for the examination of non-acute behavioral deficits produced by longer-term consequences of NMDA receptor antagonism. We examined the effects of PAOPA on acute and longer-term PCP-induced effects, including pre-pulse inhibition (PPI) deficits, hyperlocomotion, heightened brain neuronal activity, impaired novel object recognition and social interaction deficits.

## Materials and Methods

### Animals

Fifty-four male Sprague-Dawley rats were received from Charles River (Wilmington, MA, United States) at 250–300 g, and individually housed at the McMaster University Central Animal Facility (CAF). The rats were maintained on a reversed 12:12 light/dark cycle, with all testing occurring in the dark cycle. Animals were fed *ad libitum* throughout the experiment. All animal housing and testing was conducted in accordance with the Canadian Council on Animal Care and approved by McMaster University’s Animal Research Ethics Board (Animal Utilization Protocol: 14-08-28).

### Treatment Regimen

**FIGURE 1:**
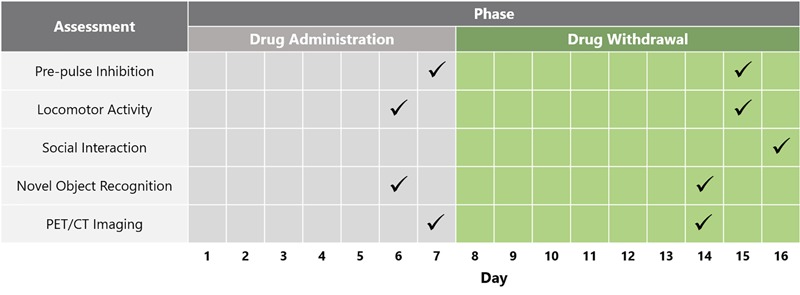
Schematic depicting timeline of behavioral and biochemical testing across the drug administration and drug withdrawal phases.

Following baseline locomotor activity and PPI testing, the animals were separated into four different treatment groups that did not differ in PPI and locomotor activity: Group (A) *vehicle* (saline) (*n* = 12); Group (B) *PCP–saline* (*n* = 15); Group (C) *PCP–PAOPA* to test the preventative effects of PAOPA (*n* = 15), and; Group (D) *one-time reversal* group to test the ability of PAOPA to reverse PCP-induced abnormalities (*n* = 12) (Table 1). PCP (Toronto Research Chemicals, Toronto, ON, Canada) and PAOPA (custom synthesized at the University of Minnesota) (Yu et al., 1988) were dissolved in 0.9% saline for each treatment. Drug solutions were prepared daily and injected intraperitoneally. PCP was injected at 5 mg/kg, PAOPA was injected at 1 mg/kg, and saline was injected at 2 mL/kg. The vehicle (Group A), PCP–saline (Group B) and PCP–PAOPA (Group C) groups received a saline (2 mL/kg) injection prior to testing to control for the PAOPA injection in the one-time reversal group. The one-time reversal group (Group D) received PCP injections similar to the PCP–saline group, but was pre-treated with a one-time PAOPA (1 mg/kg) injection before testing. The PCP–PAOPA group (Group C) received PCP and PAOPA concomitantly during the injection period. Animals were injected twice daily, 12 h apart, for 7 days. This sub-chronic PCP treatment regimen has previously been shown to induce significant schizophrenia-like behavioral deficits, while this dose of PAOPA has previously alleviated similar behavioral deficits in an amphetamine-induced model of schizophrenia (Beyaert et al., 2013; Janhunen et al., 2015). Please see Figure 1 for a schematic outlining the timing and testing protocol for this study.

**Table 1:** Outline of the treatment parameters (drug, dose and regimen) for each group according to the drug administration or drug withdrawal phase.

	Phase	
Group name	Drug administration	Drug withdrawal
Vehicle	2 mL/kg saline treatment (twice daily for 7 days)	Saline pre-treatment (once before each test)
PCP–saline	5 mg/kg PCP treatment, 2 mL/kg saline treatment (twice daily each for 7 days)	Saline pre-treatment (once before each test)
PCP–PAOPA	5 mg/kg PCP treatment, 1 mg/kg PAOPA treatment (twice daily each for 7 days)	Saline pre-treatment (once before each test)
One-time reversal	5 mg/kg PCP treatment, 2 mL/kg saline treatment (twice daily each for 7 days)	PAOPA pre-treatment (once before each test)

### Pre-pulse Inhibition

Deficits in PPI serve as a robust indicator of disrupted sensorimotor gating. In healthy subjects, a small acoustic stimulus (prepulse) given before a strong startle-eliciting stimulus (startle pulse) attenuates the response to the startle-eliciting stimulus. This ability is impaired in patients with schizophrenia since they are not able to reduce the startle reflex as effectively as control subjects when presented with a prepulse (Grillon et al., 1992; Geyer et al., 2001). Startle responses were measured using the SR-Lab Startle Response System (San Diego Instruments, San Diego, CA, United States). Each rat was habituated to the startle apparatus. PPI was tested on day 7 and day 15. For testing that occurred during the withdrawal period, the one-time reversal group received a 1 mg/kg PAOPA pre-treatment 55 min prior to testing, while the remaining groups received saline (2 mL/kg) administration. For testing, each rat was placed in the startle apparatus with a 65-decibel (dB) background white noise for a 5-min acclimatization period. Following this period, five startle pulse-alone (110 dB, 40 ms) trials were presented. Next, 65 randomized trials consisting of no pulse (0 dB), a startle pulse (110 dB, 40 ms), a pre-pulse (either 68 dB, 71 dB, or 77 dB) presented 100 ms before the startle pulse, or one of the three pre-pulses alone were presented. The startle responses were measured every 1 ms in a 100 ms period following presentation of the startle stimulus. Percent PPI was calculated using the formula: %PPI = [1 – (P + S)/S] × 100, where P + S is the mean response amplitude for pre-pulse-plus-startle pulse trials and S is the mean response amplitude for the startle pulse alone trials.

### Locomotor Activity

The behavioral phenotype of hyperlocomotor activity has been demonstrated to reflect increased dopaminergic activity in the mesolimbic system, which is associated with the positive symptomology of schizophrenia (Sams-Dodd, 1998). Locomotor activity was measured using AccuScan computerized chambers (AccuScan, Instruments, Columbus, OH, United States), which recorded multidirectional movements using photobeam breaks. Rats were habituated to the chambers before baseline testing. For baseline testing, the rats were placed in the chambers and their activity recorded for 180 min. Locomotor activity was tested on day 6, immediately following the injections, as well as on day 15, following the drug withdrawal phase. The rats were pre-treated with either saline (2 mL/kg) or PAOPA (1 mg/kg) and placed in the locomotor chambers for 50 min. Following the 50-min period, the rats were injected with a PCP challenge (5 mg/kg; 2 mL/kg) and then placed back in the locomotor chambers for 120 min. The data was then analyzed by comparing the total distance traveled among the treatment groups.

### Social Interaction

Deficits in social interaction in pre-clinical animal models of schizophrenia represent aspects of the negative symptomology of schizophrenia (Sams-Dodd, 1998, 1999). We assessed social interaction by recording the interaction between two unfamiliar rats in an open Plexiglass arena (100 cm × 75 cm × 40 cm). Rats were individually habituated to the arena before testing. On day 16, the rats were grouped into pairs within their treatment group and then colored with a non-toxic paint (red or blue). The animals were then pre-treated with either saline (2 mL/kg) or PAOPA (1 mg/kg). 55 min following the pre-treatment injection, the animals were simultaneously placed in the Plexiglass arena, in opposite corners, and left to interact for 15 min. Experimenters, blind to treatments, then scored video recordings of each rat for the number of interactions, categorized by the following types of interactions: sniffing, following, aggression, and crawling over or under another rat. The first 5 min of the interaction session were not scored and counted as habituation.

### Novel Object Recognition

The novel object recognition task provides a proxy measure for assessing aspects of cognition in rodents. This task relies on the rat’s tendency to explore novel objects over familiar objects, reflecting the rat’s memory of the latter (Rajagopal et al., 2014; Janhunen et al., 2015). Individuals with schizophrenia show deficits in object recognition tasks such as the Brief Visual Memory Test, used to assess visual learning and memory (Young et al., 2009).

Novel object recognition was tested in a Y-maze that consisted of an octagonal center platform (52 cm diameter) with two arms 70 cm long, 25.5 cm high, and 12.5 cm wide. Animals were habituated to the Y-maze prior to testing. Testing occurred on day 6, which occurred 90 min following injections and on day 14 following the drug withdrawal phase. During testing rats were placed in the maze for 3 min of habituation and then returned to their home cage for 1 min. In this 1-min delay the maze was cleaned with 75% ethanol and two identical objects were placed at the end of each arm of the maze. The rat was then placed back in the maze for 3 min while the time spent interacting with each object was recorded. The rat was then returned to the home cage for 1 min, in which the maze was cleaned again with 75% ethanol. Two objects were placed in the maze, one object in each arm: one was an identical copy of the previous objects; the other was a novel object. The objects were similar in height and volume, but differed in shape and appearance. The rat was then returned back to the maze and the time spent interacting with each object was recorded. Novel object recognition was assessed for a second time in the drug withdrawal phase on day 14, following the same protocol but with different objects. During this phase, a PAOPA (1 mg/kg) or saline (2 mL/kg) pre-treatment was administered 55 min prior to testing. Novel object recognition was analyzed using a discrimination index (DI), calculated using the formula: DI = [(Novel Object Exploration Time/Total Exploration Time) - (Familiar Object Exploration Time/Total Exploration Time)] × 100.

### Positron Emission and Computerized Tomography Imaging

PET/CT fused imaging offers a highly translatable tool to assess hypo- and hyperfrontality in live animals (Daya et al., 2014). Cerebral metabolic activity was measured with the expertise of the McMaster Centre for pre-clinical and translational imaging. To create higher-resolution images that would facilitate more precise region of interest (ROI) analysis, we combined PET and CT scans with a reference MRI.

Animals were randomly selected (*n* = 4) from each group and imaged twice (first on day 7 and second on day 14). Following administration of [18F]FDG (500 μCi in 400 μL saline) via tail vein injection, rats were placed in their home cages for 30 min, at which point they were anesthetized using 1.5% isoflurane and secured in a container designed for imaging. Animals were first placed in a Philips Mosaic dedicated Animal PET system (Philips Medical Systems, Cleveland, OH, United States) for 15 min of static emission PET scan and then a Gamma-Medica Ideas X-SPECT (Gamma-Medica Ideas, Northridge, CA, United States) for 5 min of high-resolution CT acquisition.

CT images were co-registered onto a prototypical CT and MRI scan from a healthy male Sprague-Dawley rat (450 g) which served as a template onto which all subsequent PET/CT images were co-registered. The representative CT image was co-registered to the MRI scan by maximizing the mutual information algorithm on a 3D affine transformation, using the MATLAB and the FAIR Toolkit (Modersitzki, 2009). The template CT image was completed by sizing it to the same dimensionality as the MRI scan. Next, each subject’s PET/CT fused scans were co-registered onto the template CT scan by minimizing the sum of squared differences between the subject PET/CT scans and the template CT scan. Finally, the co-registered PET/CT scan was sized to the same dimensionality as the MRI scan. Eight regions of interest (ROIs) were assessed for [18F]FDG uptake. The maximum and mean subject standard uptake value (SUV), as well as the population SUV mean, standard deviations, and σ mean, were determined for each region. Finally, the *z*-score for SUV mean for each region was calculated and compared between PCP–saline and PCP–PAOPA treated groups.

### Statistical Analysis

All statistical analyses were completed using GraphPad Prism software (GraphPad Software, San Diego, CA, United States). Because there were four different groups of treatment that differed only in treatment, a one-way analysis of variance (ANOVA) was performed with Tukey’s *post hoc* analysis for the outcomes of baseline locomotor activity (before the subchronic regimen) and novel object recognition. Significance was taken as *p* < 0.05 and outliers were identified using Grubb’s test with a significance level of α ≤ 0.05. A repeated measures two-way ANOVA with Bonferroni *post hoc* comparisons was conducted for PPI, social interaction, and locomotor activity (after the subchronic regimen) with a drug challenge (for PPI the two factors were decibel level and treatment; for social interaction, type of interaction and treatment; for locomotor activity, drug challenge and treatment). For the PET/CT imaging analysis, *Z*-scores (relative to healthy, food-restricted, vehicle treated animals) were calculated for each ROI. Values larger than 1.98 represented regions outside the 95th percentile of the normal group and were considered significant and hypermetabolic (or hypometabolic if Z was equal to or less than -1.98).

## Results

### Pre-pulse Inhibition

**FIGURE 2:**
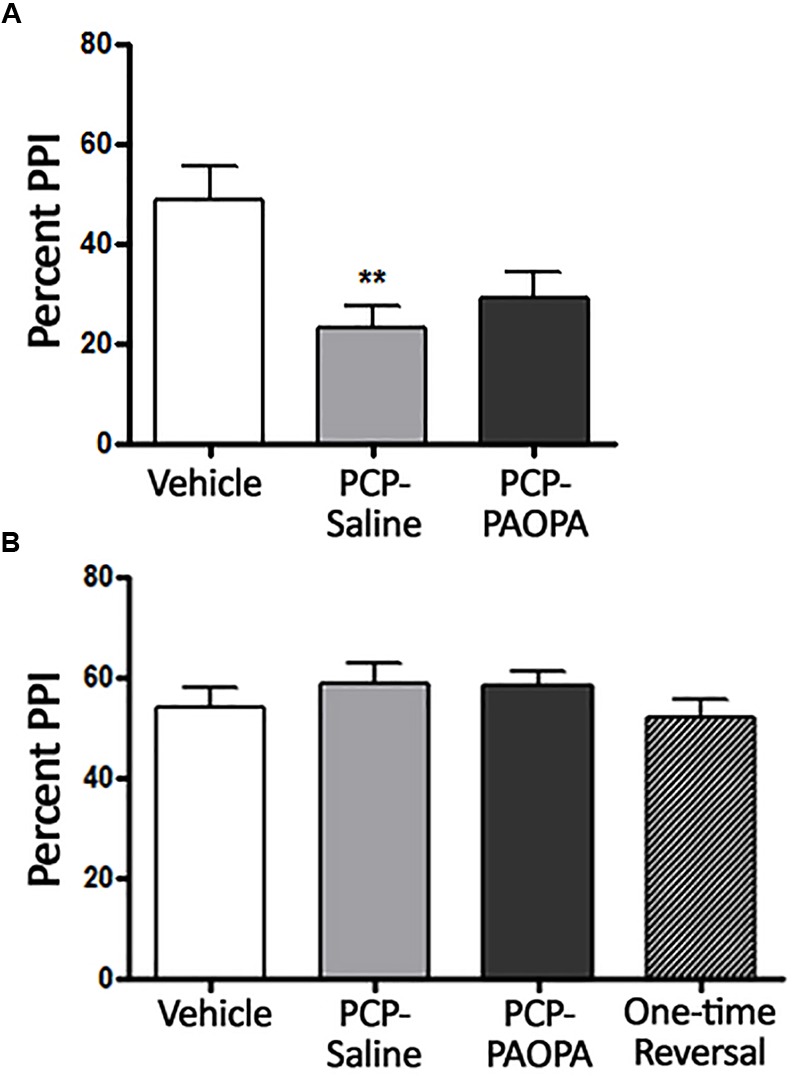
Effects of PCP treatment, concurrent PCP and PAOPA treatment, and a one-time administration of PAOPA following PCP treatment on pre-pulse inhibition (PPI) in rats. **(A)** Assessment following final drug administration (day 7) showed significantly reduced percent PPI in the PCP–saline group when compared to the vehicle group (^∗∗^*p* < 0.01). **(B)** No significant differences in percent PPI, between groups, were observed when tested during the drug withdrawal phase, 1 week after the final drug administration (day 15).

Two-way ANOVA revealed a significant (*F*_2,9_= 4.467, *p* = 0.0449) effect of treatment when rats were treated 10 min prior to PPI testing on day 7, the final day of drug administration (Figure 2A). Bonferroni’s multiple comparison post-test revealed that the PCP–saline treated group had significantly reduced (MD = 26.28, 95% CI = 13.12–39.44) PPI compared to the vehicle treated group (*p* < 0.0001). Bonferroni’s multiple comparison post-test did not reveal a significant difference between vehicle and PCP–PAOPA-treated groups (*p* > 0.05) or between PCP–saline and PCP–PAOPA treated groups (*p* > 0.05).

Following acute testing, rats were tested for PPI 1 week after final drug administration (Figure 2B). No significant main effect of treatment was observed in the two-way ANOVA (*p* > 0.05).

### Locomotor Activity

**FIGURE 3:**
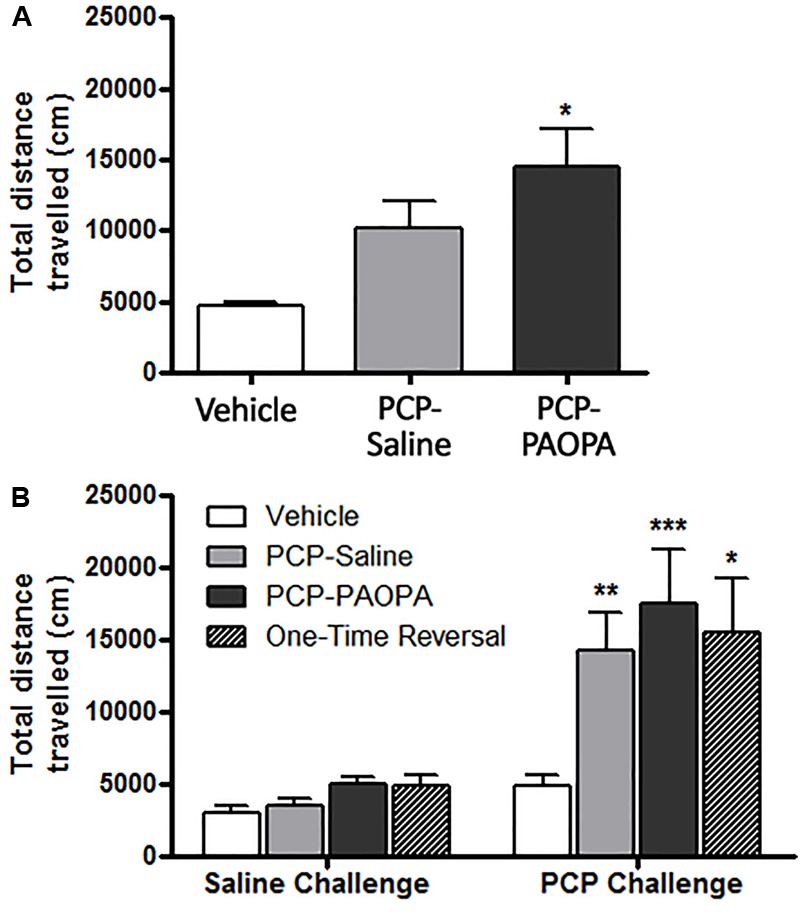
Effect of PCP treatment, concurrent PCP and PAOPA treatment, and a one-time administration of PAOPA following PCP treatment on rat locomotion. **(A)** Following initial drug administration, treatment with both PCP and PAOPA significantly increased total distance traveled compared to the vehicle group (*p* < 0.05). **(B)** PCP (5 mg/kg) challenge following 7 days of drug withdrawal (day 15) significantly increased locomotor activity compared to the vehicle group in PCP–saline, PCP–PAOPA, and one-time reversal groups (^∗∗∗^*p* < 0.001; ^∗∗^*p* < 0.01; ^∗^*p* < 0.05).

Locomotor activity was assessed after the final drug administration on day 6 and during the drug withdrawal phase on day 15. On the first day of drug administration, rats were put into locomotor activity chambers immediately after being injected. One-way ANOVA revealed a significant difference (*F*_2,33_ = 3.85, *p* = 0.0314); Tukey’s multiple comparison post-tests revealed that only the vehicle and PCP–PAOPA treatment groups differed: PCP and PAOPA appeared to enhance locomotion (MD = 9721 cm, 95% CI = 1115–18330 cm, *p* < 0.05) (Figure 3A). Prior studies have confirmed that acute PAOPA does not, on its own, produce a hyperlocomotive state; as such, we attribute the increased locomotion to the effects of PCP (Beyaert et al., 2013).

Following 8 days of withdrawal, animals were again tested for locomotor activity and administered either 2 mL/kg saline or 1 mg/kg PAOPA. Fifty minutes later, all animals were challenged with 5 mg/kg PCP. Two-way ANOVA revealed a significant main effect of the 5 mg/kg PCP challenge dose (*F*_1,32_= 29.27, *p* < 0.0001) and a significant main effect of treatment (*F*_3,32_= 3.485, *p* = 0.0270) (Figure 3B). No significant effects of interaction (*F*_3,32_= 2.431, *p* = 0.0832) or subject-matching (*F*_32,32_= 1.236, *p* = 0.2763) were observed. Bonferroni post-tests revealed that the vehicle group had lower locomotor activity than PCP–saline (MD = 9413 cm, 95% CI = 444.7–18390 cm, *t* = 3.119, *p* < 0.01), PCP–PAOPA (MD = 12600 cm, 95% CI = 3323–21880 cm, *t* = 4.036, *p* < 0.001), and one-time PAOPA reversal (MD = 10630 cm, 95% CI = -1594 – 22860 cm, *t* = 2.584, *p* < 0.05) treatment groups. Bonferroni post-tests did not reveal any significant differences between the PCP–saline and PCP–PAOPA or one-time reversal treatment groups.

### Social Interaction

**FIGURE 4:**
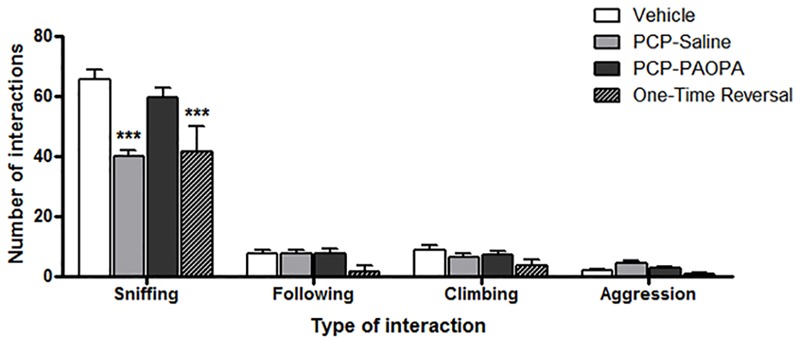
Effects of PCP treatment, concurrent PCP and PAOPA treatment, and a one-time administration of PAOPA following PCP treatment on social interaction assessed via sniffing, following, climbing, and aggressive behaviors. To represent negative symptoms of schizophrenia, interaction between two unfamiliar rats of the same treatment group was recorded following 8 days of drug withdrawal (day 16). Both PCP–saline and one-time reversal groups showed a decrease in the number of sniffing interactions compared to the vehicle group. The PCP–PAOPA group exhibited more sniffing interactions than either PCP–saline or one-time reversal groups (^∗∗∗^*p* < 0.001).

Social interaction was assessed on day 16, 8 days following cessation of drug administration (Figure 4). Two-way ANOVA revealed a significant main effect of the type of interaction recorded (*F*_3,120_= 545.2, *p* < 0.0001), treatment (*F*_3,120_= 8.486, *p* = 0.0002), subjects-matching (*F*_40,120_= 2.041, *p* = 0.0016), and interaction (*F*_9,120_= 13.28, *p* < 0.0001). Further analysis using Bonferroni post-tests revealed significant differences, specifically for sniffing and not for the other types of interactions. The vehicle group had more sniffing interactions than the PCP–saline (MD = 25.29, 95% CI = 17.56–33.01, *t* = 10.24, *p* < 0.001) and the one-time PAOPA reversal group (MD = 23.75, 95% CI = 12.16–35.34, *t* = 6.415, *p* < 0.001). Furthermore, PCP–PAOPA had more sniffing interactions than the PCP–saline (MD = 19.62, 95% CI = 11.58–27.66, *t* = 7.637, *p* < 0.001) and one-time PAOPA reversal (MD = 18.08, 95% CI = 6.286–29.88, *t* = 4.796, *p* < 0.001) groups.

### Novel Object Recognition

**FIGURE 5:**
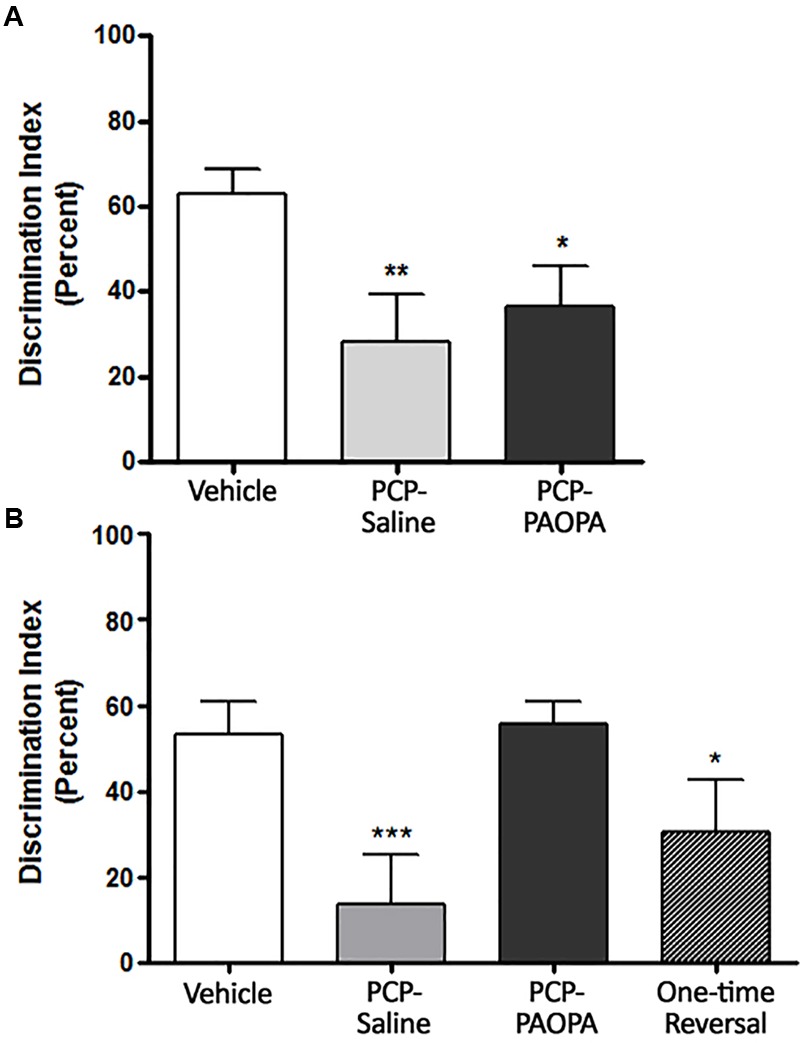
Effects of PCP treatment, concurrent PCP and PAOPA treatment, and a one-time administration of PAOPA following PCP treatment on novel object recognition (NOR) in rats. **(A)** On day 6, following drug administration, PCP–saline and PCP–PAOPA groups demonstrated a reduced discrimination index compared to vehicle treated animals (^∗∗^*p* < 0.01; ^∗^*p* < 0.05). **(B)** Following 7 days of drug withdrawal (day 14), PCP–saline and one-time reversal groups showed a significantly lower discrimination index (^∗∗∗^*p* < 0.001; ^∗^*p* < 0.01, respectively) in comparison to vehicle treated rats. Rats treated concurrently with PCP and PAOPA had a significantly higher discrimination index than PCP–saline treated rats (*p* < 0.01).

Novel object recognition was assessed on day 6, 90 min following injection (Figure 5A) and on day 14, during the drug withdrawal phase (Figure 5B). In the acute setting, one-way ANOVA revealed a significant difference between groups (*F*_2,20_= 6.591, *p* = 0.0063). Tukey’s multiple comparison test revealed that the vehicle group had a higher percent discrimination index than PCP–saline (MD = 35.86%, 95% CI = 8.352–63.37%, *p* < 0.01) and PCP–PAOPA (MD = 27.93%, 95% CI = 1.680–54.19%, *p* < 0.05). No significant differences were found between the PCP–saline and the PCP–PAOPA group (*p* > 0.05).

In the sub-chronic experiment, one-way ANOVA again revealed a significant difference between groups (*F*_3,49_ = 7.974, *p* = 0.0002). Further analysis by Tukey’s multiple comparison test revealed several significant differences. To start, vehicle-treated rats had a higher percent discrimination index than PCP–saline (MD = 47.75%, 95% CI = 17.83–77.68%, *p* < 0.001) and the one-time PAOPA reversal (MD = 35.99%, 95% CI = 2.528–69.45%, *p* < 0.05) groups, but not the PCP–PAOPA group (*p* > 0.05). Furthermore, PCP–PAOPA had a higher percent discrimination index than the PCP–saline group (MD = -42.15%, 95% CI = -73.20 to -11.09%, *p* < 0.01). Post-tests revealed no significant difference between PCP–PAOPA and one-time PAOPA reversal (*p* > 0.05).

### Brain Metabolic Activity

Brain metabolic activity was measured with PET/CT fused computation of [18F]FDG uptake with an MRI region of interest scaffold. Animals were imaged at two time points: day 7 (final day of drug administration) and day 14 (following 7 days of drug withdrawal).

**FIGURE 6:**
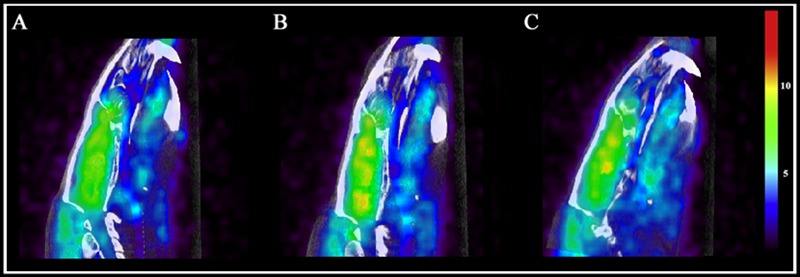
Brain metabolic activity was measured with PET/CT fused computation of [18F]FDG uptake at two time points: day 7 (final day of drug administration) and day 14 (following 6 days of drug withdrawal). Representative PET and CT fused images for **(A)** saline treated animals at day 7, **(B)** PCP treated animals at day 7, and **(C)** PCP treated animals at day 14. Color scale represents relative glucose utilization values. *Brain metabolic activity was measured via standardized uptake values with an MRI region of interest scaffold (data presented in Figure 7).*

**FIGURE 7:**
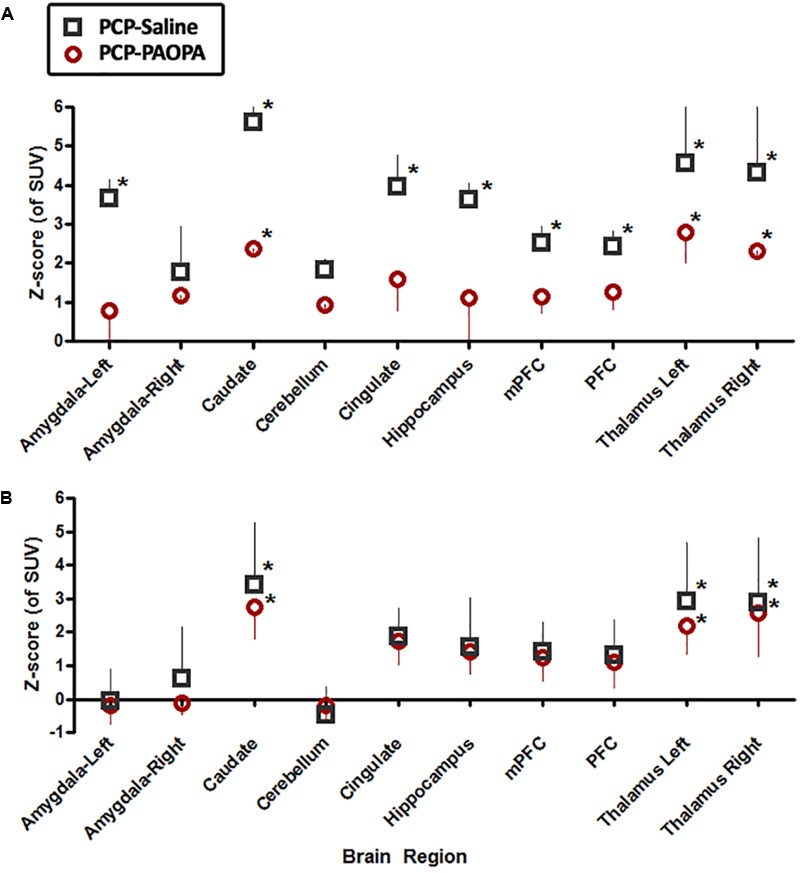
Effects of PCP-saline treatment, and concurrent PCP and PAOPA treatment on brain metabolic activity in rats. **(A)** On day 7, the final day of drug administration, both PCP–saline and PCP–PAOPA groups exhibited significantly elevated metabolic activity in the caudate and left and right thalamus in comparison to healthy rats. The PCP–saline group additionally demonstrated increased uptake in the left amygdala, cingulate, hippocampus, and left and right thalamus—PAOPA treatment appears to attenuate the hypermetabolic state in these areas. **(B)** On day 14, following 6 days of drug withdrawal, there was no significant difference in activity in the amygdala, cerebellum, cingulate, hippocampus and pre-frontal cortex of PCP–saline and PCP–PAOPA animals compared to vehicle treated animals. In the caudate and left and right thalamus both PCP–saline and PCP–PAOPA groups exhibited significantly elevated metabolic activity in comparison to healthy rats. PAOPA treatment appears to have no ameliorative effect on PCP induced brain hypermetabolic activity following drug withdrawal. ^∗^Values larger than 1.98 represented regions outside the 95th percentile of the normal group and were considered significant and hypermetabolic.

On day 7, examining the acute effects of drug treatment, PCP–saline administered animals showed significant [18F]FDG uptake in the left amygdala (*z* = 2.519), caudate (*z* = 3.706), cingulate (*z* = 2.411), hippocampus (*z* = 2.554), and left (*z* = 4.885) and right (*z* = 4.495) thalamus (Figure 6). This data demonstrates an overall pattern of cortico-limbic hypermetabolism. Although the right amygdala, cerebellum and medial prefrontal and prefrontal cortex showed an elevated metabolic response, this was not statistically significant relative to vehicle treated controls. Similarly, PCP–PAOPA treatment demonstrated, in general, a lower hypermetabolic response. PAOPA attenuated the hypermetabolic response in the left amygdala, cingulate, and hippocampus (Figure 7A). However, PAOPA was not able to mitigate the effects of PCP in the caudate and left and right thalamus.

On day 14, PCP–saline administered animals showed significant [18F]FDG uptake in the caudate (*z* = 3.706), and left (*z* = 4.885) and right (*z* = 4.495) thalamus (Figure 6). These findings demonstrate a potentially enduring increase in metabolic function in the caudate and thalamus; or conversely, a developed heightened metabolic response to drug withdrawal on day 14. The amygdala and cerebellum showed no change in brain metabolic activity relative to vehicle-treated controls (Figure 7B). The cingulate, hippocampus, medial prefrontal and prefrontal cortex, although not significantly different from vehicle-treated controls, appeared to demonstrate a moderately elevated metabolic profile (Figure 7B). PCP–PAOPA treated animals demonstrated a metabolic profile that closely mimicked PCP–saline animals, indicating PAOPA had no effect on the brain metabolic response following withdrawal.

## Discussion

This study provides further and more substantiating evidence for the therapeutic potential of PAOPA for schizophrenia. This study implicates PAOPA’s potential for treating aspects of the cognitive and negative symptoms of schizophrenia that escape improvement by current medications. PAOPA ameliorated PCP-induced social withdrawal and novel object recognition and attenuated PCP-induced hypermetabolic activity in key brain regions. PAOPA was unable to rescue acute PCP-induced PPI deficits and hyperlocomotion. These effects are contrary to PAOPA’s effects in the amphetamine-sensitized animal model of schizophrenia—a model that is predominately defined by altered dopaminergic signaling—where PAOPA ameliorated amphetamine-induced PPI deficits and hyperlocomotion (Beyaert et al., 2013). These differential effects provide insight on PAOPA’s mechanism of action and support its mediation of the positive-like symptoms via dopaminergic targets.

Concomitant treatment of PAOPA with PCP was able to significantly improve social interaction deficits produced by sub-chronic PCP treatment (Figure 4). This finding supports previous investigations of PAOPA’s ability to impede social withdrawal in sub-chronically treated MK-801 rats and amphetamine-sensitized animals (Dyck et al., 2011; Beyaert et al., 2013). MK-801, similar to PCP, is a non-competitive NMDA receptor antagonist but pharmacologically more potent than PCP. Following sub-chronic MK-801 treatment with an acute MK-801 challenge, PAOPA ameliorated MK-801-induced social-behavioral abnormalities (Dyck et al., 2011). In the amphetamine-sensitized model, PAOPA was able to both prevent (concomitant PAOPA treatment with amphetamine during sensitization; proactive) and reverse (one-time treatment with PAOPA following sensitization; retroactive) deficits in social interaction (Beyaert et al., 2013). In the present study, we observed a remedial effect with concomitant treatment of PAOPA (proactive) but not with an acute treatment of PAOPA (retroactive). Spielewoy and Markou (2003) have demonstrated that during PCP administration, rats develop a reduced brain threshold to rewarding stimuli, which increases during withdrawal from PCP. An inability to derive pleasure from rewarding stimuli may have accounted for the reduced interaction seen between PCP-treated animals. Our results suggest that concomitant treatment with PAOPA was important in attenuating social behavior deficits and the underlying neurobiological changes developed either during PCP administration or withdrawal, the latter being more likely (discussed further below).

The application of the novel object recognition task in the PCP pre-clinical model of schizophrenia has been widely established for assessing potential drug candidates for schizophrenia (Grayson et al., 2007; Meltzer et al., 2013; Rajagopal et al., 2014). Following PCP withdrawal, PAOPA (concomitant treatment with PCP) provided a proactive therapeutic effect on spatial and working memory deficits observed in the novel object recognition task (Figure 5). Jentsch et al. (1998) reported that prefrontal dopamine utilization was reduced 3 weeks after cessation of sub-chronic PCP treatment. This effect may have contributed to impairments in the novel object recognition task as dopamine activity in the frontal cortex is imperative to working memory tasks and aligns with PAOPA’s primarily dopaminergic mechanism of action (Bushnell and Levin, 1993; Jentsch and Roth, 1999; Zhang et al., 2004). In prior investigations, modafinil and lurasidone demonstrated treatment efficacy in the PCP- novel object recognition paradigm, and likewise have demonstrated pro-cognitive therapeutic effects in the clinic (Redrobe et al., 2010; Horiguchi et al., 2011). Haloperidol, risperidone, and donepezil have shown limited cognitive therapeutic efficacy in the clinic and a lack of treatment efficacy in the PCP- novel object recognition paradigm (Redrobe et al., 2010). These findings provide a compelling benchmark of PAOPA’s anticipated pro-cognitive effects.

Numerous human imaging studies utilize PET to assess brain neuronal activity either through cerebral blood flow or cerebral glucose utilization in subjects with schizophrenia. The technique employed in this study to measure brain neuronal activity (via glucose utilization) is specifically translatable to that used in the clinic, allowing us to *more* directly apply insights garnered in rats to those observed in human subjects. Recently, sub-chronic PCP administration in rats has been shown to alter brain metabolic activity as assessed by [18F]FDG, mimicking the phenomenon of hypo- or hyper-frontality observed in schizophrenia (Vollenweider et al., 1997; Pratt et al., 2008; Daya et al., 2014). Acute NMDA antagonism, assessed immediately (within 3 h) after 7 days of PCP administration, produced elevated brain activity as hypothesized and previously shown by our group and others (Gao et al., 1993; Duncan et al., 1998; Miyamoto et al., 2000; Daya et al., 2014) (Figure 6). Gao et al. (1993) and Cochran et al. (2003) describe diminished brain metabolic activity after 3 h and up to 72 h following the last administration of PCP, suggesting a biphasic effect. We next examined brain metabolic activity 7 days after sub-chronic treatment, when social and cognitive impairments maximally manifest. This study is the first to show that sub-chronic PCP treatment induces a heightened metabolic state 7 days after the last administration of PCP. This suggests that following drug withdrawal, elevated neuronal activation occurs as a result of the longer-term consequences of NMDA receptor blockade. Therefore, if a biphasic effect of PCP exists within this timeline, this effect may be a result of a compensatory response to PCP sensitization, supporting a relationship between hyperactivation—rather than hypoactivation—and social/cognitive impairment.

Findings from acute time points (day 7) and following drug withdrawal (day 14) reveal that PAOPA dampens the effects of acute NMDA blockade but not longer-term consequences of NMDA receptor antagonism. On day 7, concurrent treatment with PAOPA reduced the hypermetabolic effect of PCP, particularly in corticothalamic regions (Figure 7A). On day 14, concurrent treatment with PAOPA was unable to prevent this response (Figure 7B). Future studies should aim to examine the effect of one-time PAOPA administration on the elevated brain metabolic profile observed on day 14.

## Conclusion

In conclusion, this study revealed two important findings: (1) PAOPA mitigated deficits in social interaction and novel object recognition, and normalized heightened brain metabolic activity in the preclinical PCP animal model of schizophrenia; and, (2) Deficits in social interaction and impaired novel object recognition in the PCP animal model of schizophrenia are accompanied by significantly elevated brain metabolic activity 7 days following the last administration of PCP. Ultimately, this study further validates the therapeutic potential of PAOPA for the treatment of schizophrenia, and provides unique insight into the construct validity of the sub-chronic PCP rat model of schizophrenia.

## Author Contributions

RM and RD conceived the study. RD, JB, SK, and JH conducted the behavioral and imaging experiments. RD, CR, NB, and TF contributed to the analysis and registration of the microPET/CT imaging and MRI scaffolding. RD and JB analyzed the data, wrote the manuscript, and prepared the figures. All authors contributed to review of the manuscript.

## Conflict of Interest Statement

RM is an inventor of the intellectual property, PAOPA, and its application is secured by the US patent: PCT/CA2011/000968. The remaining authors declare that the research was conducted in the absence of any commercial or financial relationships that could be construed as a potential conflict of interest.
